# Defining a clinical protocol using a computerized central visual processing battery

**DOI:** 10.1016/j.mex.2026.103789

**Published:** 2026-01-08

**Authors:** Marcelo Fernandes Costa, Leonardo Dutra Henriques, Givago Silva Souza

**Affiliations:** a Departamento de Psicologia Experimental, Instituto de Psicologia, Universidade de São Paulo, Av. Prof. Mello Moraes 1721, Butanta, 05508-030, São Paulo, SP, Brasil; b Núcleo de Medicina Tropical, Universidade Federal do Pará, Rua Generalíssimo Deodoro, 92 – Umarizal, 6605.5240, Belém, Pará, Brasil

**Keywords:** Visual Processing Assessment, Visual Processing Disorder, Visual Perception, Global Processing, Local Processing, Dorsal Stream, Ventral Stream, Spatial Memory, Reading Span, Automatic Attention, Sustained Attention, Quantitative Estimation

## Abstract

This article presents the development and validation of a computerized clinical protocol for assessing Central Visual Processing (CVP). The protocol was designed to overcome limitations in current visual assessment tools by integrating sensory, perceptual, and cognitive visual functions within the dorsal and ventral processing streams. It comprises psychophysically controlled tasks measuring contrast sensitivity, texture perception, coherent motion, form integration, visual attention, reading-related eye movements, quantity estimation, and spatial-numerical mapping. Stimuli were developed using high-precision presentation software, and procedures were adapted to ensure both clinical feasibility and psychophysical validity.

Method validation was conducted with 41 healthy adults through test–retest analysis, Cronbach’s alpha, and Spearman–Brown split-half reliability. No significant differences were observed between first and second assessments (p > 0.05), and reliability indices showed strong internal consistency across subtests. These findings confirm the reproducibility and methodological robustness of the protocol.

• A comprehensive computerized battery assessing central visual functions across dorsal and ventral streams
• Psychophysical methods adapted for clinical precision and feasibility
• Strong reliability demonstrated through test–retest, internal consistency, and split-half correlations

## Graphical abstract

**Figure:**
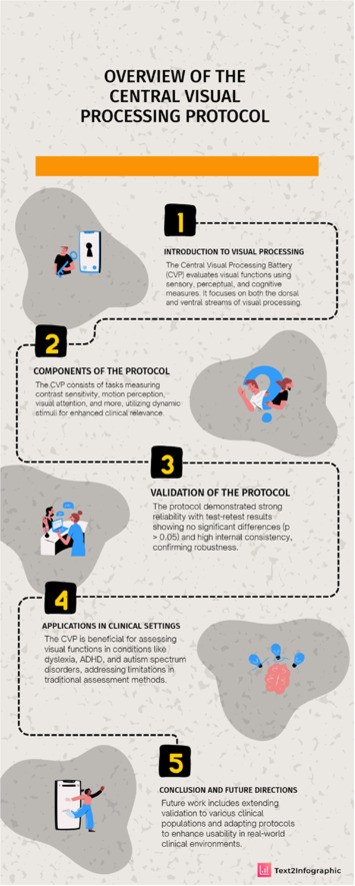

## Specifications table

**Table:** 

Subject area	Psychology
More specific subject area	Perception and Cognition
Name of your protocol	Battery for Central Visual Processing (BCVP)
Reagents/tools	The Central Visual Processing Battery was developed in Psykinematix software for visual psychophysics (version 1.6, KyberVision Japan LLC, Myagi, Japan) [1]; iMac OS X computer (version 10.8.5, intel Core i5 processor, 2.5 GHz - Cupertino, California, USA).
Experimental design	This protocol followed the methodological steps of conception, project specifications, development of clinically viable psychophysical measures, and validation of subtests in a clinical battery to assess central visual processing. Subsequently, tolerance threshold measures were defined to create normal value ranges and their clinical availability.
Trial registration	Non applicable
Ethics	All participants consented to their participation in the study. Informed assent was obtained from all the parent’s participants. The study is under the ethical standards of the 1964 Helsinki Declaration and its later amendments. The study was approved by the Instituto de Psicologia da USP ethic research committee with the number CAAE: 11,979,513.9.0000.5561.
Value of the Protocol	• This protocol consists of a computerized battery to clinically evaluate visual functions, from sensory to cognitive levels, selecting the functions affected in various conditions such as Learning Difficulties, Attention Deficit Disorder, Down Syndrome and Hyperactivity and Autism Spectrum Disorder. • This protocol is innovative in assessing dynamic aspects of visual processing, previously unavailable for clinical measurement. This is crucial, since most of the changes revealed in the literature demonstrate alterations in the dynamic aspects of visual perception and cognition. • The conceptual approach is another strength of this assessment. We shifted from a function-based approach, typically considered in neuropsychological assessments, to one based on the Dorsal and Ventral Streams of Visual Processing. This allows us to trace the origin of visual difficulties from sensory inputs from the Magnocellular and Parvocellular pathways to higher-order perceptual functions such as spatiotemporal integration, reading span, and quantity estimation.

## Background

The assessment of Central Visual Processing (CVP) is a recent clinical demand. For a long time, clinically measured visual functions have taken place—and still take place—mainly in ophthalmologic consultations, in which visual acuity and stereopsis are the most frequently assessed parameters. Depending on the case, additional clinical measures of visual functions may be requested, such as visual perimetry, luminance contrast sensitivity testing, and color vision evaluation [2]. On the other hand, assessments of perceptual and cognitive visual functions are only superficially examined in a few subtests within neuropsychological evaluation batteries. However, these assessments assume that the visual system is intact, since their primary focus lies on higher-order perceptual and cognitive measures [3]. For many years, this gap between ophthalmological measures and higher-order visual functions remained insufficiently assessed.

The bridging of this gap emerged with the development of several batteries designed to measure Visual Perception. However, there remain many important considerations that limit the applicability of these perceptual function assessment protocols, which are currently regarded primarily as screening tools. This situation arises from several critical factors. First, these assessment batteries are measurement protocols adapted directly from neuropsychological evaluations and, as such, retain many of the same limitations—such as analyses based on “isolated” functional domains [4]. They are neither adaptable to, nor do they account for, ophthalmological visual alterations, which leads to a high rate of false negatives, in addition to lacking a minimally robust standardization of administration. Second, more recent studies have shown that the internal correlations among different perceptual visual functions assessed are weak to moderate, supporting the early assumption that these batteries consist merely of separate testing blocks of visual functions with little interrelation [5]. This fragmentation hinders the understanding of the perceptual continuum between sensation and cognition. Third, some tests focus on highly specific functions, such as visuomotor interactions or spatial skills related to fine motor activity, among others [6]. Fourth, a frequent point of criticism concerns the quality of the stimuli employed, which are mostly figures presented in identification or discrimination tasks. The levels of complexity in these tasks often increase disproportionately, thereby reducing the sensitivity of the measures [7]. Fifth—and perhaps most importantly—these batteries are typically administered using printed materials, eliminating any dynamic component of visual assessment. This is a significant limitation, given that at least half of our visual functions are dynamic in nature. Furthermore, this static materials confine stimulation to very high suprathreshold levels, failing to assess performance across varying levels of brightness, contrast, and spatial resolution, which further decreases the overall sensitivity of the assessment. Finally, the last issue concerns the metric system used in these evaluations. All these batteries provide output results in the form of categorical classifications or percentile-based functional rankings. The absence of interval-scale metrics diminishes the significance and population representativeness of the results, offering little additional information beyond what is already provided by ophthalmological and neuropsychological evaluations.

The need to establish a clinical protocol for assessing Central Visual Processing (CVP) has become increasingly pressing, given the significant rise in demand for measures of visual functions associated with the growing number of clinically identified cases of Attentional Deficit and Hyperactive Disorder (ADHD), Dyslexia (DIX), Dyscalculia (DYC), high-functioning autism spectrum disorder (ASD), and Down syndrome (DS). In these populations, psych pedagogical, speech-language, and occupational therapy evaluations have consistently revealed significant impairments in visual functions—often in the presence of normal ophthalmological findings. Therefore, our protocol for developing a Central Visual Processing assessment battery aims both to address this emerging clinical demand and to overcome the limitations of the visual function measures currently available in clinical practice.

## Description of protocol

### Phase I – conceptual planning

The first phase of the protocol’s development aimed to establish a new conceptual framework for visual processing—one that moved beyond a function-based perspective to instead reflect the two major visual processing pathways that are well established in the literature: the dorsal and ventral streams [8]. In this way, the assessment protocol would be capable of evaluating, in parallel, the distinct functions processed within each pathway, as well as different levels of complexity for each function [9]. Within this framework, the inclusion of dynamic visual functions became an essential requirement.

The initial stage of visual processing must begin at the level of sensory processing. Along the progression of the ventral stream, it is essential to assess functions related to local aspects such as form, detail, and object processing at different functional levels—namely detection, discrimination, and identification. In contrast, as processing advances along the dorsal stream, it becomes necessary to evaluate contextual spatial measures, motion perception, and global spatial processing.

### Phase II – literature background

The literature review was initially guided by the specific needs expressed by the target clinical groups. The first guiding question concerned which visual functions have been empirically demonstrated to be affected in each condition. Subsequently, the scope of the review was broadened to determine whether these same functions are also compromised in other conditions. This approach allowed for the identification of the most relevant parameters for developing both the visual stimuli and the psychophysical procedures to be employed.

We identified that, in Visual Processing Disorder, alterations occur in reduced luminance contrast sensitivity [10,11], reduced low perceptual functions as textures [[12], [13], [14]], increased crowding in contextual simple form discriminations [15,16], increased subjective spatial centre imprecision and line distortions [17,18]. In Dyslexia, changes are frequently observed in visual functions related to reduced reading acquisition [19,20], reduced visual span [21,22]. In Dyscalculia, visual alterations have been found in number sense [23,24], spatial relations (Geometry) [25], quantitative manipulation [26]. In ADHD, different visual functions appear to be affected depending on the subtype: in the impulsive/inhibitory-control-reduction type, deficits are typically observed in reduced peripheric and spatial attention [27,28], reduced quantitative processing [29]; whereas in sustained attention, the most affected functions are reduced feature detection [30,31], spatial visual search [32,33]. In ASD, the functions affected are reduced face processing (Gender, Emotions, Non-verbal Language) [34], reduced inhibitory mechanisms [35], reduced motion perceptions (Coherent Motion, Biological Motion, Organic Motion) [36], reduced time, quantitative and abstract spatial processing [37]. Although the available literature is limited, in DS alterations have been reported in reduced motion perception [38], reduced spatial localization and organization [39], and reduced numerical processing [40].

### Phase III – protocol definition

#### Stimulus and presentation

Stimuli for contrast sensitivity measurements consist of sinusoidal gratings; for radial contrast sensitivity, the stimuli are concentric patterns with Bessel frequency distributions. Both types of stimuli include gratings of 0.4, 1.6, 3.2, 6.4, and 12.8 cycles per degree of visual angle (cpd) and subtend a total size of 4° of visual angle.

The temporal luminance contrast stimulus consists of a circular area subtending 2° of visual angle, temporally modulated in contrast at frequencies of 2.0, 8.0, 18.0, and 24 Hz.

The texture contrast task evaluates the modulation of a texture composed of spatial pink noise, at spatial frequencies of 0.4, 1.6, 3.2, and 6.4 cpd. In all contrast-based protocols, stimuli are presented for a duration of 3.0 s.

Coherent motion perception is analysed separately for ventral and dorsal stream influences. In this task, forty dots, each subtending 0.2° of visual angle, move at velocities of 2°/s and 10°/s within a 10° visual field for 3.0 s.

Gestalt form integration is assessed through the perception of a circle composed of 20 or fewer 3.0°-long line segments arranged in collinearity, displayed against a background of 50 randomly oriented grating segments, for a duration of 200 ms.

Shape discrimination involves identifying one geometric figure with 6 or 10 sides among 24 other polygons ranging from 6 to 10 sides, all displayed for 20 s.

Line bisection is tested using a horizontal bar of 10° visual angle intersected by a vertical bar of 1° visual angle, presented at offsets of 0.2°, 0.45°, and 0.60° to the right or left of the true centre.

Automatic attention is evaluated using a 3.0°-wide grating oriented at 95°, 110°, or 120° among a field of 99 gratings oriented at 90°, displayed for 200 ms.

Sustained attention in a conjunction task involves orientation and luminance polarity: a 1° bar appears for 3.0 s among 21, 35, or 50 distractors.

Saccadic planning for reading is assessed through the identification of syllables first presented at the centre of the visual field and subsequently to the right at eccentricities of 0.4°, 2.0°, and 6.8° The first syllable appears for 120 ms, followed immediately by the second syllable, displayed for either 120 ms or 240 ms.

Visual quantity judgment is evaluated by comparing two sets of dots: one set of 40 dots appears to the left of fixation, while the other, displayed to the right, contains 15, 25, 30, or 38 dots.

Finally, the mental number line is assessed using a horizontal line 30° long and 1° thick, intersected by a vertical bar of 1° visual angle. A number between 2 and 9 (excluding 5) appears in the upper corner of the display. Both are presented for 30 s.

#### Psychophysical methods

Our protocol comprises two types of measurement approaches. The first involves precise psychophysical measurements, based on threshold determination. Psychophysical staircase procedures were employed for this purpose. For a clinical measurement protocol, a major challenge lies in defining a procedure that is both as efficient and as precise as those used in experimental settings, which typically require many reversals—around eleven—to determine reliable thresholds. Psychophysical staircase procedures were therefore adapted to achieve equivalent threshold values and the same criterion level (79.4 %), but with substantially fewer reversals—only four. In this adapted method, correct responses result in a decrease in the stimulus intensity, magnitude, or orientation, whereas incorrect responses lead to an increase. Traditionally, these adjustments occur in symmetrical and proportional step sizes (e.g., ±10 %). To expedite the procedure, asymmetric progressions were implemented so that the target criterion could be reached more rapidly, using the PEST (Parameter Estimation by Sequential Testing) method. Accordingly, for the first two reversals, the stimulus magnitude is reduced by 50 %, while for the final four reversals, the reduction is 12.5 %. Errors consistently increase the value of the subsequent stimulus by 25 %.

The second type includes performance-based tasks, which rely on the method of constant stimuli within a psychophysical framework. The psychophysical fitting in this method is simpler, as performance data are used to fit a psychometric function, which serves as the basis for result interpretation. For the tasks employing the psychophysical method of constant stimuli, stimulus values were adjusted to ensure a minimal number of repetitions sufficient to achieve a reliable fit of the psychometric function. A summary of the protocol is presented in Table 1.

**Table 1:** Summary of the protocol of CVP evaluation for clinical application.

Sub Test	Stimulus	Variable Details	Psychophysical Method
Linear Grating Contrast Sensitivity	linear grating, size 4°, Gabor function envelope, 5s	SF: 0.4, 1.6, 3.2, 6.4, 12.8 cpd	Staircase method with 6 reversals
Radial Grating Contrast Sensitivity	radial grating, size 4°, Gabor function envelope, 5s	SF: 0.4, 1.6, 3.2, 6.4, 12.8 cpd	Staircase method with 6 reversals
Temporal Contrast Sensitivity	Luminance modulation, size 4°, Gabor function envelope 5s	TF: 2.0, 8.0, 18.0, 24.0 Hz	Staircase method with 6 reversals
2nd Order Contrast Sensitivity (Texture)	linear grating, pink noise carrier, size 4°, Gabor function envelope	SF: 0.4, 1.6, 3.2, 6.4 cpd	Staircase method with 6 reversals
Coherent Motion	50 dots of 0.3° in coherence motion to left or to the right	permanency 100 ms 1000 ms, vel.: 2°/s, 10°/s	Staircase method with 6 reversals
Line Bisection	horizontal line of 15°, horizontal black marker 0.1°	posit: -0.60°, -0.45°, -0.20°, 0.0, 0.20°, 0.45°, 0.60°	Stimulus Constant Method, 7 jugdments
Form Detection Colinear Perception	linear grating 3.5cpd, size 0.5°, diameter 4° and 7°	30 distractors, random orientation, 6 to 20 collinear	Staircase method with 6 reversals
Form Distortion	radial circle 4° diameter, thickness 0.1°, 0.3°	radial deformity 5dpc, 10dpc	Staircase method with 6 reversals
Form Discrimination	24 geometric figures (distrctors), 1 figure (target), size 1°	Distractors: 6, 7, 8, 9, 10 sides, Target: 6, 10 sides	Stimulus Constant Method, 7 jugdments
Subjective Time Perception	circle 1°, 5° leftside, rightside	target: duration 0.8 s, 1.2 s, 1.6 s, 2.1 s; ref: 1.0s	Stimulus Constant Method, 7 jugdments
Automatic Attention	linear grating 3.5cpd, size 0.5°, oriented 90°	99 distractros, 1 target: oriented 100°, 110°, 125°	Stimulus Constant Method, 7 jugdments
Sustained Attention	1° bars, duration 3 s, orientation: black 0,0°, 90°, white 90°	target 1 white bar 0.0°, n° distractors 21, 35, or 50	Stimulus Constant Method, 7 jugdments
Quantitative Estimation	ref.: 40 dots 0.3°, 4° area, leftside, target: rightside	target: 15, 25, 30, 38 dots	Stimulus Constant Method, 7 jugdments
Mental Number Line	horizontal line 30° long, 1° thick, intending 0 to 10 scale, 20s	numbers tested: 2, 3, 4, 6, 7, 8, 9, mouse position x-axis	Free mouse position on x-axis
Saccadic Planning for Silabic Reading	syllabes ref.: ZU, MA, VI, TA, LO, target: SA, PA, BA, NA, RA, DA, FA	first central 120 ms, second 120 m 240 ms, 0.4°, 2.0°, 6.8°	Stimulus Constant Method, 7 jugdments
Rapid Automated Naming	naming, 7 numbers set, radomly chosen between 0–9	presentation 500 ms, 950 ms, 1400ms	3 trials, Reaction Time mean

SF: spatial frequency; TF: temporal frequency; cpd: cicles per degree; dpc: deformity per circle; HZ: Hertz; s.: seconds; vel.: velocity; posit.: position; ref: reference.

### Protocol validation

The validation of the protocol was analysed through replicability and reliability measures and split-half correlation, based on pilot data collected from 41 healthy adult participants. These participants were recruited by convenience sampling from among students and staff at the University of São Paulo, with a mean age of 20 years (SD = 2 years), 22 females. Replicability was analyzed using a test–retest procedure for all 41 participants. The retest was conducted after a minimum interval of two weeks (mean = 22 days, SD = 5 days). The order of the subtests was randomized for each participant, based on a predefined randomization table. The analysis of variance revealed no statistically significant differences between the first and second assessments for any of the subtests evaluated (p > 0.05), with a large effect size indicated by eta squared (η² = 0.417). The correlation between first and second measurements were high correlated (Table 2).

**Table 2:** Correlation coefficents between first and second measurments.

Sub Test	Variables						
**Linear Grating Contrast Sensitivity**	**0.4**	**1.6**	**3.2**	**6.4**	**12.8**		
	r= 0.567	r= 0.929	r= 0.855	r= 0.811	r= 0.612		
**Radial Grating Contrast Sensitivity**	**0.4**	**1.6**	**3.2**	**6.4**	**12.8**		
	r= 0.507	r= 0.945	r= 0.764	r= 0.751	r= 0.497		
**Temporal Contrast Sensitivity**	**2.0**	**8.0**	**18.0**	**24.0**			
	r= 0.957	r= 0.984	r= 0.985	r= 0.936			
**2nd Order Contrast Sensitivity (Texture)**	**0.4**	**1.6**	**3.2**	**6.4**			
	r= 0.994	r= 0.989	r= 0.988	r= 0.975			
**Coherent Motion**	**0.1 - 1.5**	**0.1 - 10.0**	**0.9 - 1.5**	**0.9 - 10.0**			
	r= 0.983	r= 0.987	r= 0.977	r= 0.990			
**Line Bisection**	**-0.45°**	**-0.2°**	**0.0°**	**0.2°**	**0.45°**	**0.6°**	
	r= 0.966	r= 0.947	r= 0.855	r= 0.911	r= 0.746	r= 0.766	
**Form Detection Colinear Perception**	**4°**	**10°**					
	r= 0.628	r= 0.607					
**Form Distortion**	**5 - 0.125°**	**10 - 0.125°**	**5 - 0.500°**	**10 - 0.500°**			
	r= 0.982	r= 0.976	r= 0.987	r= 0.981			
**Form Discrimination**	**6**	**10**					
	r= 0.923	r= 0.894					
**Subjective Time Perception**	**0.8s**	**1.3s**	**1.6s**	**2.1s**			
	r= 0.897	r= 0.831	r= 0.818	r= 0.956			
**Automatic Attention**	**0.4°**	**2.0°**	**6.8°**				
	r= 0.974	r= 0.978	r= 0.831				
**Sustained Attention**	**20**	**35**	**50**				
	r= 0.972	r= 0.990	r= 0.977				
**Quantitative Estimation**	**15**	**25**	**30**	**38**			
	r= 0.951	r= 0.983	r= 0.708	r= 0.713			
**Mental Number Line**	**2**	**3**	**4**	**6**	**7**	**8**	**9**
	r= 0.924	r= 0.907	r= 0.903	r= 0.977	r= 0.987	r= 0.957	r= 0.993
**Saccadic Planning for Silabic Reading**	**0.4°**	**2.0°**	**6.8°**				
	r= 0.896	r= 0.912	r= 0.844				
**Rapid Automated Naming**	**Color**	**Number**					
	**0.500s**	**0.950s**	**1.400s**	**0.500s**	**0.950s**	**1.400s**	
	r= 0.832	r= 0.795	r= 0.866	r= 0.860	r= 0.902	r= 0.963	

The reliability was assessed using Cronbach’s alpha coefficient, which measures the internal consistency and reliability of the obtained results. We also checked the reliability using the Spearman-Brown split-half correlation, thus, if the sum scale is perfectly reliable, we would expect that the two halves are also perfectly correlated (Table 3).

**Table 3:** Spearman-Brown split-half correlation coefficents of reliability.

Sub Test	Chronbach's alpha	Split-Half Correlation		
**Linear Grating Contrast Sensitivity**	**0.904**	**0.951**		
**Radial Grating Contrast Sensitivity**	**0.873**	**0.915**		
**Temporal Contrast Sensitivity**	**0.927**	**0.977**		
**2nd Order Contrast Sensitivity (Texture)**	**0.862**	**0.896**		
**Coherent Motion**	**0.616**	**0.809**		
**Line Bisection**	**0.862**	**0.867**		
**Form Detection Colinear Perception**	**0.832**	**0.844**		
**Form Distortion**	**0.626**	**0.848**		
**Form Discrimination**	**0.801**	**0.819**		
**Subjective Time Perception**	**0.803**	**0.894**		
**Automatic Attention**	**0.832**	**0.875**		
**Sustained Attention**	**0.922**	**0.955**		
**Quantitative Estimation**	**0.997**	**0.995**		
**Mental Number Line**	**0.893**	**0.937**		
**Saccadic Planning for Silabic Reading**	**0.865**	**0.879**		
**Rapid Automated Naming**	**Number**	**Color**	**Number**	**Color**
	**0.842**	**0.716**	**0.901**	0.818

The validation of the Central Visual Processing Battery (CVP) demonstrated strong methodological robustness across multiple indices of reliability. Test–retest analysis, conducted with 41 healthy adults, showed no statistically significant differences between the first and second assessments for any subtest (p > 0.05), indicating high temporal stability of the measures. The large effect size observed (η² = 0.417) further supports the consistency of the protocol, while the randomized subtest administration order ensured that reliability was not confounded by task sequence or learning effects. Together, these results confirm that the protocol produces stable outputs across sessions and is suitable for clinical and research settings requiring reproducible measurements.

Internal consistency metrics further corroborated the reliability of the protocol. Cronbach’s alpha coefficients, calculated across the full set of subtests, demonstrated strong internal coherence, indicating that the battery captures interrelated but distinct components of central visual processing. Complementing this, the Spearman–Brown split-half correlations also revealed high reliability, confirming that different halves of the battery yield convergent results and reinforce its structural validity. Altogether, these validation outcomes support the protocol’s capacity to measure central visual processing functions with precision, stability, and methodological integrity.

## Limitations

Despite the strong reliability and careful methodological construction of the CVP, several limitations of the protocol should be acknowledged. First, the validation sample consisted exclusively of healthy young adults recruited through convenience sampling. Although suitable for establishing baseline reliability, this demographic homogeneity limits generalizability to broader clinical populations, including children, older adults, and individuals with neurological, developmental, or ophthalmological conditions. Future research should therefore extend validation to diverse clinical cohorts to confirm diagnostic sensitivity and population-specific norms.

Second, while the protocol includes both threshold-based psychophysical measures and performance-based tasks, all validations were conducted under standardized laboratory conditions, using controlled luminance and calibrated hardware. This raises uncertainties about how the battery performs in variable clinical environments where ambient lighting, screen properties, and patient compliance may differ from experimental standards. Additional studies examining ecological validity and hardware variability are warranted to confirm robustness outside laboratory settings.

Finally, the battery demands sustained attention, consistent fixation, and accurate psychophysical responses across multiple subtests. Although these requirements are necessary for fine-grained measurement, they may impose challenges for certain clinical populations such as young children, individuals with attentional impairments, and patients with reduced cognitive flexibility. Adaptations—including shorter variants, task-specific practice trials, or automated difficulty adjustments—may be required to optimize usability for these groups.

## Related research article

***For a published article:***

Costa, M.F.; HENRIQUES, L.D.; PINHO, O.C. Development of a Spatio-Temporal Contrast Sensitivity Measurement for Clinical Testing. Journal Of Ophthalmic & Vision Research, v. 17, p. 69-77, 2022. doi:10.18502/jovr.v17i1.10172.

## Supplementary material *and/or* additional information [OPTIONAL]

No supplementary material.

## CRediT authorship contribution statement

**Marcelo Fernandes Costa:** Conceptualization, Methodology, Software, Investigation. **Leonardo Dutra Henriques:** Software, Data curation, Writing – original draft, Visualization, Investigation. **Givago Silva Souza:** Validation, Formal analysis, Writing – review & editing.

## Declaration of competing interest

The authors declare that they have no known competing financial interests or personal relationships that could have appeared to influence the work reported in this paper.

## Data Availability

Data will be made available on request.
